# Evaluating the Utility of Combined Bladder Cancer Biomarkers, the Molecular Prognostication of Tumor Subtypes, or What Else Is Needed to Illuminate Our Vision?

**DOI:** 10.3390/ijms21249657

**Published:** 2020-12-18

**Authors:** Thorsten H. Ecke, Thomas Otto, Florence Le Calvez-Kelm

**Affiliations:** 1 Department of Urology, HELIOS Hospital Bad Saarow, DE-15526 Bad Saarow, Germany; 2 Department of Urology, Rheinland Klinikum Neuss, DE-41464 Neuss, Germany; totto@lukasneuss.de; 3 International Agency for Research on Cancer (IARC), F-69372 Lyon, France; lecalvezf@iarc.fr

In the last few years, we published two special issues devoted to highlighting important scientific results in the field of bladder cancer research and clinical implications. We are now pursuing our efforts towards third edition. Bladder cancer is still one of the most frequent malignancies worldwide [1] and while many studies focused on the discovery of bladder cancer biomarkers, opinions still widely diverge on their clinical utility. Novel markers or combinations of existing tumor markers could significantly contribute to more precise diagnosis and tumor subclassifications, as well as facilitating therapeutic decision-making. The classification of bladder cancer tumors based on grade and stage alone is suboptimal in predicting the biological behavior and thus in guiding the choice of treatment, especially in high risk cases [2,3,4].

Cystoscopy and imaging systems are still considered as gold standard for the detection and monitoring of bladder cancer as they have shown unequal combined overall sensitivity and specificity. They have, however, only limited sensitivity in detecting small lesions of the urinary tract. For such cases urine cytology is still the most widely used non-invasive test for the detection and surveillance of bladder cancer. Despite its high specificity, with around 86%, the limitation of this method lies in its low sensitivity of approximately 50% [5], especially in low-grade tumors [6,7]. To date, with the absence of reliable cost-effective urinary biomarkers, the confirmation of suspected carcinomas of the urinary tract and the subsequent life-long surveillance for relapse is still being undertaken by cystoscopic examinations, which represents a significant cost burden on healthcare systems [8]. 

While none of the bladder cancer markers are recommended by international guidelines in bladder cancer management, urine cytology is still recommended for diagnostic purposes [9]. Besides the interesting protein based tumor markers, such as UBC^®^ *rapid* test, BTA stat, and NMP22 [10,11,12], there are also promising genetic based fast tests, such as the uromonitor^®^ [13]. UroMuTERT [14] and ddPCR assays [15], which are based on the detection in urine samples of TERT promoter mutations, the most common somatic mutations in bladder cancer [16]. All of these markers have demonstrated acceptable to high sensitivities in all or in some subgroups of bladder cancer. Interestingly, TERT promoter mutations have been recently shown to be detectable in urinary DNA samples of asymptomatic individuals years prior to primary diagnosis of bladder cancer with high specificity, demonstrating its potential as simple non-invasive biomarkers for early detection [15]. Studies comparing different fast tests to the old gold standard, urine cytology based on the Paris system for reporting [17] and integrating clinical parameters such as hematuria, ECOG performance score, smoking behavior and others [18] are needed.

The molecular subtyping of bladder cancer has been well accepted after its initial introduction in 2014 [19,20,21]. Especially muscle-invasive tumors have been categorized into basal and luminal subtypes such as molecular breast cancer subtypes originally described by Perou et al. [22], which were subsequently shown to be predictive of clinical outcomes. The molecular prognostication of breast cancer is likely to be transposable to bladder cancer. In this line, basal types of muscle invasive bladder cancer have been shown to be associated with shorter disease-specific and overall survival, presumably because patients with these cancers tended to have more invasive and metastatic disease at presentation [20].

There is an urgent and tremendous need for clinical markers to predict recurrence and progression of bladder cancer; these markers likely contributing to establish better personalized treatments. Molecular staging of urological tumors will allow selecting cases that will require systemic and/or target treatment [23,24].

Keeping all that in mind we should perceive that marker systems are playing an important role in all fields of bladder cancer: as an alternative or complement to cystoscopy during post-surgery surveillance for monitoring relapse, as predictor and prognostic tool during decisions for systemic therapies or as screening tool for detecting bladder cancer in high-risk groups.

This Special Issue has been introduced with the aim of offering the possibility to publish new research results in the field of bladder cancer basic and translational research. While editing this Special Issue, we have appreciated that significant progress has been recently made in bladder cancer research and that efforts should be pursued by fostering extensive cooperation between the scientific and medical communities to translate evidence-based research into clinical practice. The identification and validation of bladder cancer markers for predicting recurrence and progression will contribute establishing better treatments tailored to the individual patient based on their predicted response. Molecular staging of urological tumors will allow selecting cases that will require systemic treatment.

The editors thank all submitting authors for their efforts and time spent for each manuscript. The lead editor would like to thank all editors for the time spent in reviewing, assigning reviews, and commenting on submitted manuscripts. As the editorial team, we hope that this Special Issue will prove useful in planning future bladder cancer research studies.
